# The association of prediabetes and type 2 diabetes with hippocampal subfields volume: The Maastricht study

**DOI:** 10.1016/j.nicl.2023.103455

**Published:** 2023-06-22

**Authors:** Jennifer Monereo-Sánchez, Jacobus F.A. Jansen, Sebastian Köhler, Martin P.J. van Boxtel, Walter H. Backes, Coen D.A. Stehouwer, Abraham A. Kroon, Jeroen P. Kooman, Casper G. Schalkwijk, David E.J. Linden, Miranda T. Schram

**Affiliations:** a School for Mental Health & Neuroscience, Faculty of Health, Medicine and Life Sciences, Maastricht University, The Netherlands; b Department of Radiology & Nuclear Medicine, Maastricht University Medical Center, The Netherlands; c Department of Psychiatry and Neuropsychology, Maastricht University Medical Center, The Netherlands; d Alzheimer Centrum Limburg, Department of Psychiatry and Neuropsychology, School for Mental Health and Neuroscience, Faculty of Health, Medicine and Life Sciences, Maastricht University, The Netherlands; e School for Cardiovascular Diseases, Faculty of Health, Medicine and Life Sciences, Maastricht University, The Netherlands; f Department of Internal Medicine, Maastricht University Medical Center, The Netherlands; g School of Nutrition and Translational Research in Metabolism, Maastricht University, Maastricht, The Netherlands; h Maastricht Heart+Vascular Center, Maastricht University Medical Center, The Netherlands

**Keywords:** Brain atrophy, Glucose metabolism, Hippocampal subfields, MRI, Prediabetes, Type 2 diabetes

## Abstract

**Aims/hypothesis:**

We investigated whether prediabetes, type 2 diabetes, and continuous measures of hyperglycemia are associated with tissue volume differences in specific subfields of the hippocampus.

**Methods:**

We used cross-sectional data from 4,724 participants (58.7 ± 8.5 years, 51.5% women) of The Maastricht Study, a population-based prospective cohort. Glucose metabolism status was assessed with an oral glucose tolerance test, and defined as type 2 diabetes (n = 869), prediabetes (n = 671), or normal glucose metabolism (n = 3184). We extracted 12 hippocampal subfield volumes per hemisphere with FreeSurfer v6.0 using T1w and FLAIR 3T MRI images. We used multiple linear regression and linear trend analysis, and adjusted for total intracranial volume, demographic, lifestyle, and cardiovascular risk factors.

**Results:**

Type 2 diabetes was significantly associated with smaller volumes in the hippocampal subfield fimbria (standardized beta coefficient ± standard error (β ± SE) = -0.195 ± 0.04, p-value < 0.001), the hippocampus proper, i.e. Cornu Ammonis (CA) 1, CA2/3, CA4, dentate gyrus, subiculum and presubiculum (β ± SE < -0.105 ± 0.04, p-value < 0.006); as well as the hippocampal tail (β ± SE = -0.162 ± 0.04, p-value < 0.001). Prediabetes showed no significant associations. However, linear trend analysis indicated a dose–response relation from normal glucose metabolism, to prediabetes, to type 2 diabetes. Multiple continuous measures of hyperglycemia were associated with smaller volumes of the subfields fimbria (β ± SE < -0.010 ± 0.011, p-value < 0.001), dentate gyrus (β ± SE < -0.013 ± 0.010, p-value < 0.002), CA3 (β ± SE < -0.014 ± 0.011, p-value < 0.001), and tail (β ± SE < -0.006 ± 0.012, p-value < 0.003).

**Conclusions/interpretation:**

Type 2 diabetes and measures of hyperglycemia are associated with hippocampal subfield atrophy, independently of lifestyle and cardiovascular risk factors. We found evidence for a dose–response relationship from normal glucose metabolism, to prediabetes, to type 2 diabetes. Prediabetes stages could give a window of opportunity for the early prevention of brain disease.

## Highlights

• Type 2 diabetes is associated with smaller volumes in most hippocampal subfields.
• Prediabetes shows no significant associations with any hippocampal subfield volume.
• There is a dose–response trend from normal glucose metabolism to type 2 diabetes.
• Prediabetes might offer a window of action for the early prevention of brain disease.

## 1 Introduction

There is extensive evidence that type 2 diabetes is associated with an increased risk of both degenerative and vascular brain damage (Brundel et al., 2014, Zheng et al., 2018), as well as with memory impairment (Callisaya et al., 2019, Sadanand et al., 2016). Given the involvement of the hippocampus in memory processing, the relation between type 2 diabetes and hippocampal atrophy has been widely studied. Most brain MRI studies have indeed detected an association between type 2 diabetes and smaller bilateral hippocampal volume (Cui et al., 2019, Gold et al., 2007, Hempel et al., 2012, Moran et al., 2013). Although there are exceptions (Wisse et al., 2014), a later *meta*-analysis based on 1,364 cases and 3,433 controls confirmed the association between type 2 diabetes and smaller hippocampal volumes (Moulton et al., 2015).

The hippocampus is a heterogeneous structure, composed of multiple subfields, each of which is characterized by specific cellular composition and distinctive neurophysiology (Fanselow and Dong, 2010). Therefore, type 2 diabetes pathophysiology may be differently associated with specific hippocampal subfields. Yet, there is little theoretical agreement on the hippocampal subfields that might be affected in type 2 diabetes, and previous studies found smaller volumes in different subfields (Blom et al., 2020, Li et al., 2020a, Li et al., 2020b, Zhang et al., 2021).

Another very relevant question to the disease course of type 2 diabetes is whether prediabetes (i.e., the intermediate hyperglycemic condition in the transition from normal glucose metabolism to type 2 diabetes) is also associated with smaller hippocampal volumes. Previous literature found no evidence of an association between prediabetes and total hippocampal volume (THV) (Marseglia et al., 2019, Schneider et al., 2017). However, whether there is an association between prediabetes and specific hippocampal subfields is still unknown.

The aim of the current study is to investigate whether prediabetes, type 2 diabetes, and continuous measures of hyperglycemia are associated with lower hippocampal subfield volumes. In addition, we aim to investigate whether potential associations are independent of demographic, lifestyle, and cardiovascular risk factors. To our knowledge, no previous study addressed the association between prediabetes, type 2 diabetes, and hippocampus subfield volumes in a population-based cohort, and taking into account potential confounders.

## 2 Material and methods

### 2.1 Study population and design

**Fig. 1:**
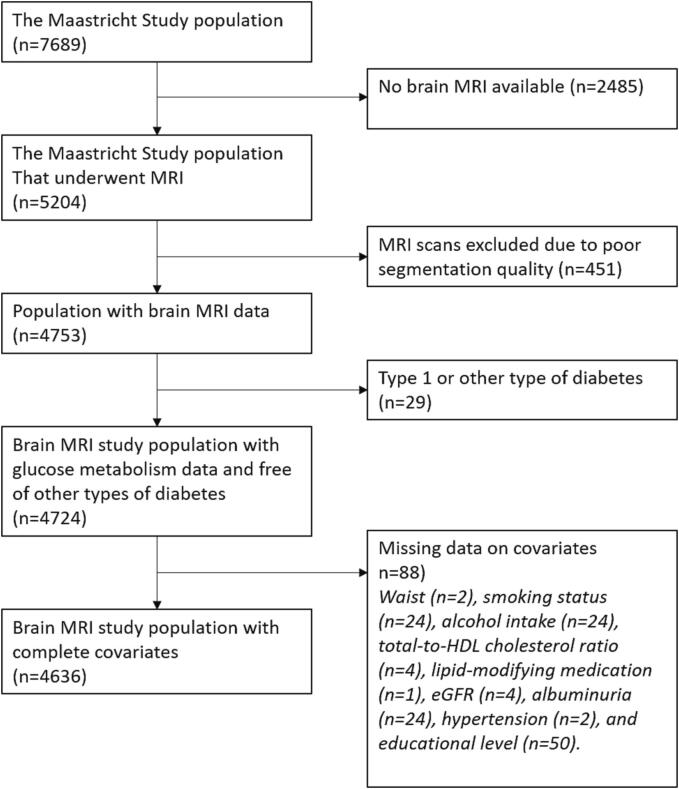
Flowchart of the study population.

We used data from The Maastricht Study, an observational population-based cohort study. The rationale and methodology have been described previously (Schram et al., 2014). In brief, the study focuses on the etiology, pathophysiology, complications and comorbidities of type 2 diabetes, and is characterized by an extensive phenotyping approach. Eligible for participation were all individuals aged between 40 and 75 years and living in the southern part of the Netherlands. Participants were recruited through mass media campaigns, the municipal registries, and the regional Diabetes Patient Registry via mailings. Recruitment was stratified according to known type 2 diabetes status, with an oversampling of individuals with type 2 diabetes, for reasons of efficiency. The present report includes cross-sectional data from 7,689 participants, who completed the baseline survey between November 2010 and December 2017. The examinations of each participant were performed within a time window of three months. MRI measurements were implemented from December 2013 onwards until February 2017 and were available in 5,204 out of 7,689 participants. Additionally, 451 MRI scans had insufficient segmentation quality (Monereo-Sánchez et al., 2021). Participants with type 1 diabetes or other types of diabetes (n = 29) were excluded from the analysis. In the remaining 4,724 participants, complete data on covariates was available in 4,636 participants (Fig. 1). The study has been approved by the institutional medical ethical committee (NL31329.068.10) and the Minister of Health, Welfare and Sports of the Netherlands (Permit 131088-105234-PG). All participants gave written informed consent.

### 2.2 Glucose metabolism status

To determine glucose metabolism status, all participants, except those who used insulin, underwent a standardized 2 h 75 g oral glucose tolerance test (OGTT) after an overnight fast. For safety reasons, participants with a fasting plasma glucose level above 11.0 mmol/l, as determined by a finger prick, did not undergo the OGTT (n = 50). For these individuals, fasting plasma glucose level and information about diabetes medication use were used to determine glucose metabolism status. Glucose metabolism status was defined according to the World Health Organization 2006 criteria as normal glucose metabolism (fasting plasma glucose < 6.1 mmol/l), prediabetes (fasting plasma glucose ≥ 6.1 and < 7.0 mmol/l) or type 2 diabetes (fasting plasma glucose ≥ 7.0 mmol/l, or the use of diabetes medication) (Schram et al., 2014).

### 2.3 Brain imaging

Brain images were acquired on a 3T magnetic resonance scanner (MAGNETOM Prismafit, Siemens Healthineers GmbH) located at a dedicated scanning facility (Scannexus, Maastricht, The Netherlands) using a head/neck coil with 64 elements for parallel imaging. The MRI protocol included a three-dimensional (3D) T1-weighted (T1w) magnetization prepared rapid acquisition gradient echo (MPRAGE) sequence (repetition time/inversion time/echo time (TR/TI/TE) 2,300/900/2.98 ms, 176 slices, 256 × 240 matrix size, 1.0 mm cubic reconstructed voxel size); and a fluid-attenuated inversion recovery (FLAIR) sequence (TR/TI/TE 5,000/1,800/394 ms, 176 slices, 512 × 512 matrix size, 0.49 × 0.49 × 1.0 mm reconstructed voxel size).

Brain segmentation was performed with FreeSurfer v6.0 (Fischl, 2012) using T1w and FLAIR images as input. The optional arguments “FLAIRpial” and “3T” were used to optimize segmentation quality. Brain segmentations with insufficient quality, i.e. Euler numbers below 1.5 quartile (-80 for left hemisphere and −68 for right hemisphere) were excluded (Monereo-Sánchez et al., 2021). Hippocampal subfields were segmented (Iglesias et al., 2015), yielding a THV and 12 regions of interest per hemisphere. Subfields name and description can be found in Supplementary Table S1. Hippocampal subfields volumes were averaged between the left and right hemisphere of each participant. Supplementary table S1 shows the mean volume and standard deviation of each subfield across the reference sample (n = 4724), which were used for z-transformation prior to statistical analysis. Results are depicted in hippocampal subfield maps, a legend for these maps can be found in Supplementary Fig. S1.

### 2.4 General characteristics and covariates

As described elsewhere (Schram et al., 2014), educational level (low, intermediate, high), alcohol intake, smoking status (never, current, former) and history of cardiovascular disease were assessed by questionnaires. Medication use was assessed in a medication interview where generic name, dose, and frequency were registered. We measured weight, height, body mass index, waist circumference, office and ambulatory 24 h blood pressure, plasma glucose levels, serum creatinine, 24 h urinary albumin excretion (twice), hemoglobin A1c (HbA1c), and plasma lipid profile as described elsewhere (Schram et al., 2014). Estimated glomerular filtration rate (in ml/min/1.73 m2) was calculated with the Chronic Kidney Disease Epidemiology Collaboration equation based on both serum creatinine and serum cystatin C (Inker et al., 2012).

### 2.5 Statistical analysis

All statistical analyses were performed by use of R 4.0.2 statistical software (2020–06–22). General characteristics of the study population were presented as mean with standard deviation, or as percentages, and were evaluated by T-tests or χ^2^ tests.

We used multiple linear regression analysis to investigate the association of prediabetes, type 2 diabetes, HbA1c, fasting plasma glucose, and 2 h post-load glucose levels with THV and hippocampal subfield volumes. Analyses were performed for THV and each hippocampal subfield (n = 13 brain volume estimates). Analyses were adjusted for age, sex, total intracranial volume and the time between the baseline and MRI measurement, waist circumference, smoking status, alcohol intake, total‐to‐high-density lipoprotein (HDL) cholesterol ratio, lipid-modifying medication, estimated glomerular filtration rate (eGFR), albuminuria, hypertension, and educational level. Given THV and 12 subfields were analyzed, and to maintain a type I error rate of 5%, Matrix Spectral Decomposition (Nyholt, 2004) was used to determine the effective number of independent variables. Based on the resulting eigenvalues, the obtained effective number was n = 7, therefore alpha threshold for significance was set at 0.05/7 = 0.0071.

In post-hoc analysis, we tested for a linear trend using the ordinal variable of glucose metabolism status (normal glucose metabolism = 1, prediabetes = 2, and type 2 diabetes = 3). This analysis was justified after checking the model fit with the main model (glucose metabolism status as a categorical measure). Comparison of the log likelihood ratio’s showed a better fit with the ordinal variable, which indicates a dose–response relationship between glucose metabolism status and the hippocampal subfield volumes.

Several additional analyses were performed to check for robustness. To study whether the associations found in continuous measures of hyperglycemia (i.e. HbA1c, fasting plasma glucose, or 2 h post-load glucose levels) were driven by the oversampling of individuals with diagnosed type 2 diabetes, we additionally excluded participants with type 2 diabetes from the analyses. To address whether the hippocampal volume differences are independent from general brain atrophy, we replaced total intracranial volume with total brain volume. We also replaced waist circumference with body mass index, and total-to-HDL cholesterol ratio for low-density lipoprotein (LDL) cholesterol level. Additionally, we report the results without the exclusion of cases with insufficient quality segmentation based on Euler numbers. Finally, an interaction term was incorporated to test for interaction among prediabetes, type 2 diabetes, and continuous measures of hyperglycemia and sex, on hippocampal subfield volumes.

## 3 Results

### 3.1 General characteristics of the study population

General characteristics of the study population, stratified by glucose metabolism status, are shown in Table 1. The study population consisted of 4724 participants; 3184 participants had normal glucose metabolism, 671 had prediabetes, and 869 had type 2 diabetes. The mean age was 58.7 ± 8.5 years, and 51.5% were female. Participants with prediabetes and type 2 diabetes were older, less often female, had a worse cardiovascular risk profile, were more often current smokers, and more often had a low educational level (Table 1). Mean subfields volumes can be found in Supplementary Table S1. Individuals who underwent MRI were younger, were less likely to have type 2 diabetes, were less often current smokers and less often had a low educational level, as compared to the study population which did not undergo MRI (Supplementary Table S2).

**Table 1:** General characteristics of the study population.

Characteristic	Normal glucose metabolism (n = 3184)	Prediabetes (n = 671)	Type 2 diabetes (n = 869)	P-value
Demographics				
Age (years)	57.3 ± 8.5	61.3 ± 7.9	61.6 ± 7.9	<0.001
Sex (% female)	56.8	46.1	36.0	<0.001
Education level, low/medium/high (%)	26.6/28.5/44.9	36.4/27.7/35.8	41.2/28.9/29.9	<0.001
Glucose metabolism				
Fasting glucose (mmol/l)	5.1 ± 0.4	5.8 ± 0.6	7.7 ± 1.8	<0.001
2 h post‐load glucose (mmol/l)	5.3 ± 1.1	8.2 ± 1.7	14.2 ± 4.0	<0.001
HbA1c (mmol/mol)	35.1 ± 3.9	37.8 ± 4.4	50.0 ± 10.9	<0.001
Cardiovascular risk factors				
Waist circumference (cm)	89.7 ± 11.0	96.9 ± 12.0	103.0 ± 12.5	<0.001
Office systolic blood pressure (mmHg)	129.2 ± 16.2	135.4 ± 16.5	140.1 ± 16.9	<0.001
Office diastolic blood pressure (mmHg)	74.7 ± 9.7	76.6 ± 9.6	77.2 ± 9.4	<0.001
Hypertension (%)	36.8	59.6	77.8	<0.001
LDL (mmol/l)	3.3 ± 0.9	3.3 ± 1.0	2.5 ± 0.9	<0.001
Total‐to‐HDL cholesterol	3.5 ± 1.1	3.9 ± 1.3	3.7 ± 1.2	<0.001
eGFR (ml/min/1.73 m2)	77.5 ± 12.9	77.1 ± 14.1	81.2 ± 18.8	<0.001
History of CVD (%)	9.5	12.7	17.5	0.654
Albuminuria, micro/macroalbuminuria (%)	3.9/0.3	5.4/0.3	15.5/1.2	<0.001
Medication use				
Antihypertensive medication (%)	19.6	39.8	64.3	<0.001
Lipid‐modifying medication (%)	13.4	28.2	67.5	<0.001
Life style factors				
Smoking, never/former/current (%)	42.5/45.8/11.7	34.9/53.4/11.7	35.2/50.8/14.00	0.0106
Alcohol intake, none/low/high (%)	14.5/60.2/25.3	17.0/56.7/26.3	26.0/56.0/17.9	<0.001
Brain MRI characteristics				
Estimated total intracranial volume (mm^3^)	1472257.1 ± 148293.9	1455241.6 ± 138958.9	1455750.8 ± 138233.5	<0.001
Brain volume (mm^3^)	1191747.4 ± 116151.6	1171788.1 ± 113658.1	1160039.5 ± 111192.3	<0.001
MRI lag time (years)	1.2 ± 1.3	1.3 ± 1.3	1.3 ± 1.3	<0.001

Data are presented as mean ± standard deviation or percentage, and stratified for glucose metabolism status, i.e. normal glucose metabolism, prediabetes and type 2 diabetes. HbA1c indicates hemoglobin A1c; LDL: low-density lipoprotein; HDL, high‐density lipoprotein; eGFR, estimated glomerular filtration rate; CVD, cardiovascular disease.

### 3.2 Associations of prediabetes and type 2 diabetes with hippocampal volume

**Fig. 2:**
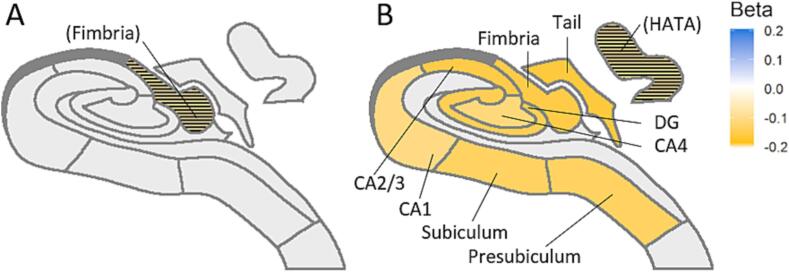
Schematic hippocampal representation displaying the associations of A) Prediabetes and B) Type 2 diabetes with hippocampal subfield volumes. Yellow color represents a negative association, i.e. Type 2 diabetes is associated with smaller subfields volume. Associations that did not survive multiple comparison correction but have P <.05 are represented with a stipe pattern and are written in parenthesis. Non-significant associations are represented in gray. See Supplementary Fig. S2 for a legend of the hippocampal map. Abbreviations: CA: Cornu Ammonis; DG: Dentate gyrus; HATA: Hippocampal-amygdalar transition area. (For interpretation of the references to color in this figure legend, the reader is referred to the web version of this article.)

Fig. 2 shows the resulting associations of THV and hippocampal subfields with prediabetes and type 2 diabetes. Detailed results can be found at Supplementary Table S3. We found no direct significant associations between prediabetes and hippocampal subfield volumes after correction for multiple comparison.

After full adjustment, type 2 diabetes was significantly associated with smaller volumes in THV (β ± SE = -0.151 ± 0.04, p-value < 0.001) as compared to normal glucose metabolism. In addition, type 2 diabetes was significantly associated with smaller volumes in the hippocampal fimbria (β ± SE = -0.195 ± 0.04, p-value < 0.001), hippocampus proper, i.e. dentate gyrus, CA1, CA2/3, CA4, subiculum and presubiculum (β ± SE < -0.105 ± 0.04, p-value < 0.006); as well as subfield tail (β ± SE = -0.162 ± 0.04, p-value < 0.001). Further, type 2 diabetes was associated also with the hippocampal-amygdala transition area (HATA, β ± SE = -0.098 ± 0.04, p-value = 0.015), although this was no longer significant after correction for multiple comparison.

In linear trend analysis there was a significant association between glucose metabolism status and smaller THV, fimbria, Cornu Ammonis (CA) 2/3, CA4, dentate gyrus, subiculum, presubiculum, and tail volumes (standardized beta coefficient ± standard error (β ± SE) < -0.054 ± 0.02, p-for-trend < 0.003, Supplementary Table S4). These results indicate a dose–response relation from normal glucose metabolism, to prediabetes, to type 2 diabetes with lower hippocampal subfield volumes.

### 3.3 Associations of continuous measures of hyperglycemia with hippocampal volume

**Fig. 3:**
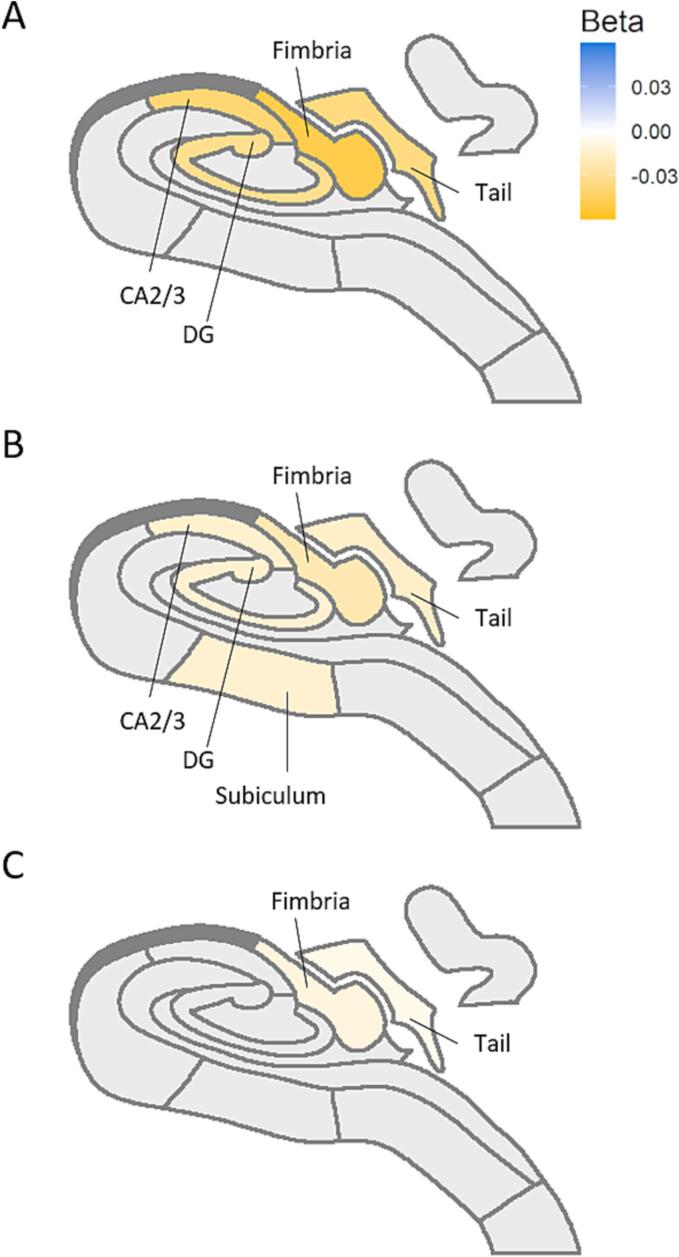
Schematic hippocampal representation displaying the significant subfields’ associations with continuous measures of hyperglycemia: **A)** Fasting glucose (mmol/l), **B)** 2 h post‐load glucose (mmol/l) and **C)** HbA1c (mmol/mol). Yellow color represents a negative association; i.e. higher values of hyperglycemia are associated with smaller hippocampal volumes. Only those structures significant after multiple comparison correction are depicted. Hippocampal subfields with no significant associations are represented in gray. See Supplementary Fig. S2 for a hippocampal map legend. Abbreviations: CA: Cornu Ammonis; DG: Dentate gyrus. (For interpretation of the references to color in this figure legend, the reader is referred to the web version of this article.)

HbA1c, fasting plasma glucose, and 2 h post-load glucose levels were associated with smaller volumes of the total hippocampus (β ± SE < -0.005 ± 0.01, p-value < 0.002), fimbria (β ± SE < -0.010 ± 0.011, p-value < 0.001), and tail (β ± SE < -0.006 ± 0.012, p-value < 0.003) after full adjustment. Fasting plasma glucose, and 2 h post-load glucose levels were additionally associated with lower volumes of dentate gyrus (β ± SE < -0.013 ± 0.010, p-value < 0.002), and CA3 (β ± SE < -0.014 ± 0.011, p-value < 0.001). 2 h Post-load glucose level was associated with lower volumes of the subiculum (β ± SE = -0.011 ± 0.004, p-value = 0.005). Results are depicted in Fig. 3. Detailed results can be found in Supplementary Table S5.

### 3.4 Additional analyses

When we limited the analysis to participants with normal glucose metabolism and prediabetes (i.e., when we excluded participants with type 2 diabetes), the significant associations between continuous measures of hyperglycemia and hippocampal subfields previously found showed numerically similar trends for the estimates, although most associations became not significant. Yet, the association between 2 h/post load glucose and smaller volumes in fimbria remained significant (Supplementary Table S6).

When we replaced estimated intracranial volume with total brain volume, the associations between type 2 diabetes and hippocampal subfields showed similar estimates, although they became not significant, with the exception of fimbria (Supplementary Table S7).

When we replaced body mass index with waist circumference the results remained consistent (Supplementary Table S8).

When we replaced total-to-HDL cholesterol ratio with LDL cholesterol level, the associations between type 2 diabetes and hippocampal subfields showed similar estimates. Although some estimates attenuated, the associations remained statistically significant (Supplementary Table S9).

The associations between prediabetes and type 2 diabetes and hippocampal subfield volumes when including the cases with low segmentation accuracy based on Euler numbers, remained consistent, and became even slightly stronger (Supplementary Table S10).

## 4 Discussion

In this study we evaluated the association of prediabetes and type 2 diabetes, as well as continuous measures of hyperglycemia, with hippocampal subfields volumes after adjustment for demographics, lifestyle, and cardiovascular risk factors. Through this large population-based study we confirmed that type 2 diabetes is associated with smaller THV, and smaller volumes in the subfields fimbria, dentate gyrus, CA1 to CA4, subiculum, presubiculum, and tail. We found no significant associations of prediabetes with THV or hippocampal subfield volumes. However, the results for continuous measures of hyperglycemia and the analysis for trend on glucose metabolism status suggest there is a dose–response association between glucose metabolism and lower hippocampal subfield volumes.

Consistent with previous literature, our results show that type 2 diabetes is associated with smaller volumes in dentate gyrus CA1, CA4, and subiculum (Blom et al., 2020, Li et al., 2020b, Zhang et al., 2021). To our knowledge, we are the first to additionally find an association of type 2 diabetes with smaller volumes in fimbria, CA2/3, and tail volumes. The standardized effect sizes of this associations ranged between −0.105 in CA1, and −0.195 in fimbria. In volume, this translates into CA1 being in average 4.22 mm^3^ smaller, and fimbria being 8.98 mm^3^ smaller in participants with type 2 diabetes when compared to those with normal glucose metabolism status. Taking into consideration the mean volume of this structures, CA1 was 1.22% smaller, and fimbria were 5.3% smaller in participants with type 2 diabetes when compared to those with normal glucose metabolism. We also found a significant association between type 2 diabetes and smaller THV, which is in line with a *meta*-analysis by Moulton et al. in 2015 (Moulton et al., 2015). Type 2 diabetes status was most strongly associated with fimbria volume, which also remained associated after correction for general atrophy of the brain. The fimbria forms a white matter bundle structure that connects the hippocampus with the rest of the brain. Smaller volumes in this specific structure could be due a loss in myelin, since white matter microstructural abnormalities (Reijmer et al., 2013) and demyelination (Li et al., 2021) has been commonly found in participants with type 2 diabetes.

In line with previous studies (Marseglia et al., 2019, Schneider et al., 2017), prediabetes showed no significant associations with hippocampal subfield volumes. However, the analysis for trend demonstrated a dose–response relationship between glucose metabolism status and lower volumes of most hippocampal subfields. This suggest there is a graded association from normal glucose metabolism to prediabetes to type 2 diabetes. Further evidence for a linear association is provided by the continuous measures of hyperglycemia, i.e., fasting plasma glucose, 2 h post-load glucose, and HbA1c levels. Specifically, continuous measures of hyperglycemia were associated with smaller volumes in THV, fimbria, dentate gyrus, CA2/3, and tail. HbA1c shows the least strong associations among the three measures, likely because it is a treatment target in type 2 diabetes. A study by Dong et al. (2019) showed associations of HbA1c with smaller volumes in dentate gyrus subiculum, and tail (Dong et al., 2019), while another study found associations with the CA1 to CA4 (Zhang et al., 2021), and dentate gyrus (Dong et al., 2019, Zhang et al., 2021). Yet, those studies observed an association of HbA1c with the molecular layer that was not detected in our study. It is important to notice that these studies used selected study populations and small sample sizes (Dong et al., 2019) (Zhang et al., 2021).

To assess robustness of the observed associations we performed a range of sensitivity analyses. First, to ensure that the observed associations would not be driven by participants with type 2 diabetes, we repeated the analysis after excluding participants with type 2 diabetes. Results were consistent with the main analysis, although regression coefficients were generally attenuated due to reduced sample size (from n = 4724 to n = 3855). However, two hours post-load glucose remained significantly associated with fimbria volume, indicating that fimbria could be one of the most sensitive subfields to hyperglycemia, and therefore bringing evidence towards subfields specificity. Second, we corrected for total brain volume instead of total intracranial volume. With this analysis we found that type 2 diabetes is associated with a smaller volume of the subfield fimbria, independent of generalized atrophy of the brain. This indicates that this subfield is affected by hyperglycemia on a higher degree than the rest of the parenchyma. Quality control of the hippocampal segmentation was provided by the exclusion of cases with outliers based on Euler numbers following guidelines (Monereo-Sánchez et al., 2021). We performed a sensitivity analysis without the exclusion of these cases. Including cases with low segmentation quality to the sample resulted in decreased p-values. This could be explained by the increase in sample size, but it might also be due to the fact that least healthy participants tend to have worse scans and poorer segmentations, with missing parenchyma in the segmentations and therefore smaller volumes.

Our results may show some specificity for subfields, as some associations with type 2 diabetes were stronger than others. In addition, several analyses using both continuous and categorical definitions of glycaemia, as well as all the sensitivity analysis allow us to detect some subfields that might have a more severe or earlier vulnerability to hyperglycemia. Both continuous measures of glycaemia as well as type 2 diabetes are consistently associated with fimbria, dentate gyrus, CA2/3, subiculum, and tail subfield volumes. These exact subfields also show increased regional vulnerability to age (Pereira et al., 2014), which agrees with the hypothesis that type 2 diabetes can be considered accelerated aging.

Strengths of this study include the large sample size and population-based design with an oversampling of type 2 diabetes; the use of oral glucose tolerance tests to accurately characterize glucose metabolism status; and the extensive phenotyping which allowed us to adjust for major cardiovascular risk factors reducing the change of residual cofounding.

This study has some limitations. The hippocampus is a small structure, and the segmentation of the hippocampal subfields is challenging and can be subject to inaccuracies. Yet, the hippocampal volumes were extracted using FreeSurfer v6.0, which shows a good manual segmentation agreement and test–retest reliability (Tae et al., 2008). We additionally improved the segmentation accuracy by adding FLAIR images for Multispectral segmentation improving segmentation reliability (Iglesias et al., 2015, Seiger et al., 2021); finally we performed quality control by the exclusion of outliers based on Euler numbers following current recommendations (Monereo-Sánchez et al., 2021). In addition, due to the population-based nature of our cohort, our results may be subject to some selection bias because participants of cohort studies are in general more health conscious. Previous research show that this can result in over- or under-estimations of the associations under investigation (Szklo and Nieto, 2014). Further, it is important to notice that the cross-sectional design of our study does not allow to claim any causality. Yet, longitudinal studies have previously shown increased rates of brain atrophy (Kooistra et al., 2013, Samaras et al., 2014) and brain function impairment (Thambisetty et al., 2013) over time in participants with impaired glucose metabolism. This suggest that glucose metabolism might affect hippocampal volumes, but future research on longitudinal data is needed to specifically address this question.

In conclusion, type 2 diabetes was associated with generalized hippocampal atrophy, which was independent of demographics, cardiovascular, and lifestyle risk factors. The fimbria is the subfield that shows the strongest association with type 2 diabetes. Continuous measures of hyperglycemia, and analysis for trend indicate that the association between hyperglycemia and hippocampal subfields volumes is linear, and follows a dose–response curve, although we could not demonstrate significant associations of prediabetes with hippocampal subfield volumes. The latter could mean prediabetes stages represent a window of action for the early prevention of brain disease.

## Funding

This study was supported by the European Regional Development Fund via OP-Zuid, the Province of Limburg, the Dutch Ministry of Economic Affairs (grant 31O.041), Stichting De Weijerhorst (Maastricht, The Netherlands), the Pearl String Initiative Diabetes (Amsterdam, The Netherlands), the Cardiovascular Center (CVC, Maastricht, the Netherlands), CARIM School for Cardiovascular Diseases (Maastricht, The Netherlands), CAPHRI Care and Public Health Research Institute (Maastricht, The Netherlands), NUTRIM School for Nutrition and Translational Research in Metabolism (Maastricht, the Netherlands), Stichting Annadal (Maastricht, The Netherlands), Health Foundation Limburg (Maastricht, The Netherlands), School for Mental Health & Neuroscience (Maastricht, Netherlands), and by unrestricted grants from Janssen-Cilag B.V. (Tilburg, The Netherlands), Novo Nordisk Farma B.V. (Alphen aan den Rijn, the Netherlands), and Sanofi-Aventis Netherlands B.V. (Gouda, the Netherlands).

## Author contributions

J.M.S., J. J., M.S., D.L. contributed to the study concept and design; J.M.S. performed the data analysis, results interpretation, and drafted the manuscript; J. J., M.S., D.L. supervised the project; J. J., M.S., D.L., S.K., M.B., W.B., C.S., A.K., J.K., C.S. contributed to the results interpretation and revision of the manuscript.

## Declaration of Competing Interest

The authors declare that they have no known competing financial interests or personal relationships that could have appeared to influence the work reported in this paper.

## Data Availability

Data will be made available on request.
