# Supplementary material for: The association of prediabetes and type 2 diabetes with hippocampal subfields volume: The Maastricht study
Source: Neuroimage Clin. 2023 Jun 22;39:103455. doi: 10.1016/j.nicl.2023.103455 (PMC10310479; doi:10.1016/j.nicl.2023.103455)
Supplement: Supplementary data 1 [file mmc1.docx]

**Supplementary material**

**Supplementary Table S1:** List of hippocampal subfields

**Supplementary Figure S1:** Hippocampal subfields map

**Supplementary Table S2:** General characteristics of the participants stratified for study inclusion

**Supplementary Table S3:** Associations of prediabetes and type 2 diabetes with hippocampal subfields.

**Supplementary Table S4:** Post-hoc analysis, linear associations of glucose metabolism status as a continuum with hippocampal subfield volumes

**Supplementary Table S5:** Associations of continuous measures of hyperglycemia with hippocampal subfield volumes

**Supplementary Table S6:** Supplementary analysis, exclusion of participants with type 2 diabetes

**Supplementary Table S7:** Supplementary analysis, substitution of intracranial volume for total brain volume

**Supplementary Table S8:** Supplementary analysis, substitution of waist circumference (cm) for Body Mass Index (kg/m2)

**Supplementary Table S9:** Supplementary analysis, total-to-HDL cholesterol ratio for LDL cholesterol level.

**Supplementary Table S10:** Supplementary analysis, report of the main results without the exclusion of cases based on MRI segmentation quality

**Supplementary Table S1 |** List of hippocampal subfields

| Abbreviation | Description | Volume mm^3^  (mean ± SD) |
| --- | --- | --- |
| THV | Total Hippocampal Volume | 3422.75 ± 349.78 |
|  |  |  |
| HATA | Hippocampus-amygdala-transition-area | 61.73 ± 9.60 |
| Fimbria | Fimbria | 75.30 ± 21.64 |
| Fissure | Hippocampal fissure | 81.42 ± 14.73 |
| Molecular layer | Molecular layer of the hippocampus | 361.43 ± 45.95 |
| Dentate gyrus | Granule cell and molecular cell layer of the dentate gyrus | 333.67 ± 38.98 |
| CA4 | Cornu Ammonis 4 | 275.04 ± 30.15 |
| CA2/3 | Cornu Ammonis 2 and 3 | 240.05 ± 36.75 |
| CA1 | Cornu Ammonis 1 | 724.29 ± 85.54 |
| Subiculum | Subiculum | 469.41 ± 50.82 |
| Presubiculum | Presubiculum | 298.92 ± 36.79 |
| Parasubiculum | Parasubiculum | 61.40 ± 11.17 |
| Tail | Hippocampal tail | 521.53 ± 67.50 |

Table shows the abbreviation and complete name for total hippocampal volume and each hippocampal subfield provided as output by FreeSurfer v.6.0, as well as the mean volume (mm^3^) ± standard deviation (SD) of the used sample (n=4724).

**Supplementary Figure S2**| Hippocampal subfields map


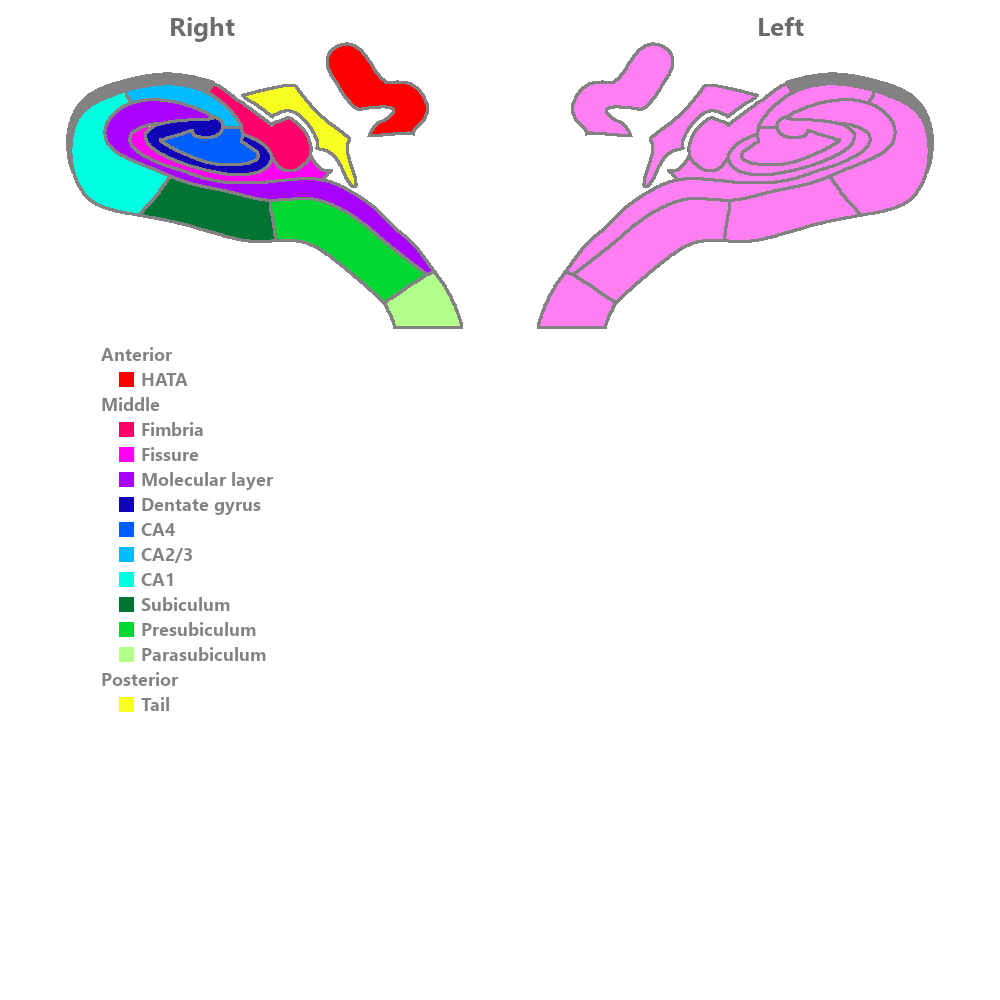

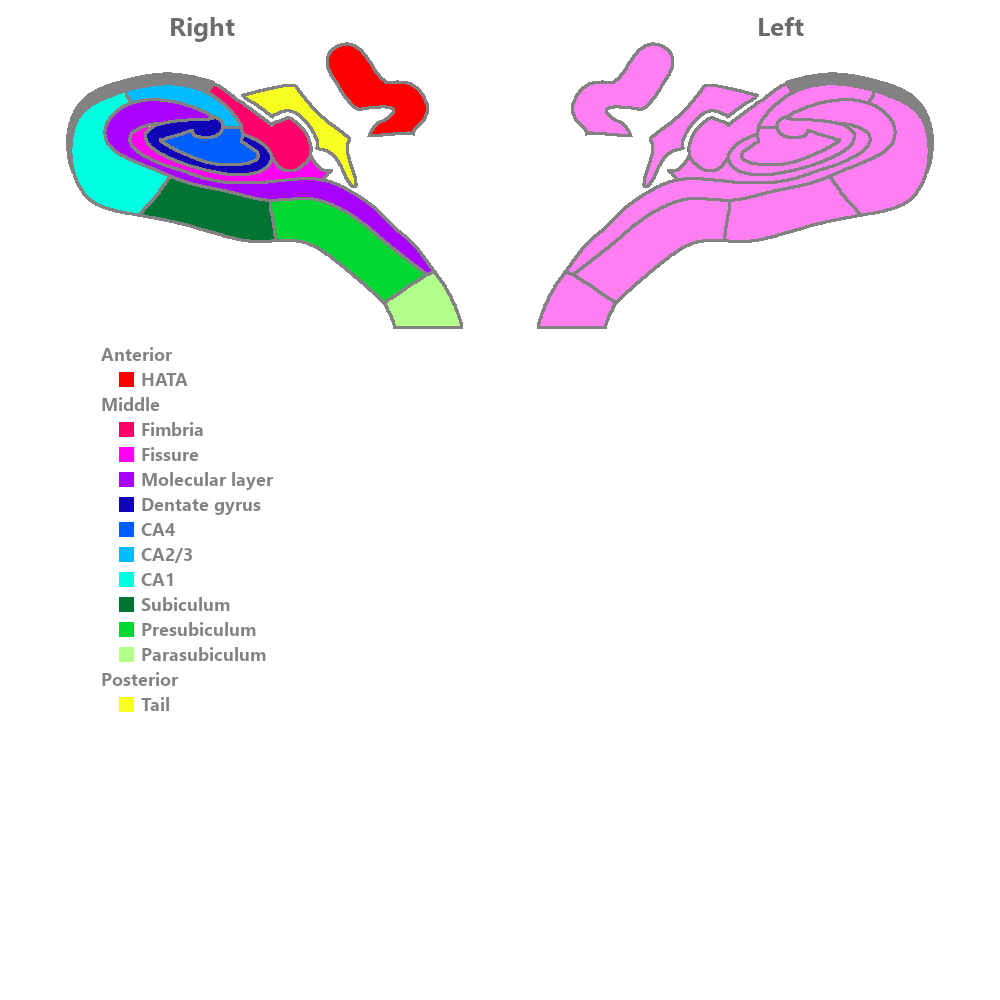


Figure shows a schematic representation of the right hippocampus in a coronal section. Hippocampal subfields are represented in different colors. *Abbreviations*: HATA, Hippocampus-amygdala-transition-area; Dentate gyrus, Granule cell and molecular cell layer of the dentate gyrus; CA, Cornu Ammonis. Notice that the subfield HATA is transposed from an anterior coronal section, while the tail is transposed from a posterior coronal section.

**Supplementary Table S2 |** General characteristics of the participants stratified for study inclusion

| Characteristic | Excluded  (n=3053) | Included  (n=4636) | P value |
| --- | --- | --- | --- |
| Demographics |  |  |  |
| Age (years) | 61.6 ± 8.6 | 58.7 ± 8.5 | <0.001 |
| Sex (% female) | 46.7 | 51.5 | <0.001 |
| Education level, low/medium/high (%) | 41.3/26.2/32.5 | 30.7/28.5/40.9 | 0.594 |
| Glucose metabolism |  |  |  |
| Status, NGM/prediabetes/T2D (%) | 48.7/16.1/35.1 | 67.4/14.2/18.4 | 0.003 |
| Fasting glucose (mmol/l) | 6.3 ± 2 | 5.7 ± 1.3 | <0.001 |
| 2h post‐load glucose (mmol/l) | 8.5 ± 4.4 | 7.1 ± 3.7 | <0.001 |
| HbA1c (mmol/mol) | 42.3 ± 11.3 | 38.3 ± 8.2 | <0.001 |
| Cardiovascular risk factors |  |  |  |
| Waist circumference (cm) | 98.8 ± 14.7 | 93.2 ± 12.6 | <0.001 |
| Office systolic blood pressure (mmHg) | 136.5 ± 19.1 | 132 ± 16.9 | <0.001 |
| Office diastolic blood pressure (mmHg) | 75.8 ± 10 | 75.4 ± 9.7 | 0.128 |
| Hypertension (%) | 65.4 | 47.6 | 0.128 |
| Total‐to‐HDL cholesterol | 3.7 ± 1.2 | 3.6 ± 1.2 | 0.015 |
| eGFR (ml/min/1.73m2) | 77.9 ± 17.6 | 78.1 ± 14.4 | 0.594 |
| History of CVD (%) | 26.2 | 11.5 | 0.594 |
| Albuminuria, micro/macroalbuminuria (%) | 10.3/1.2 | 6.2/0.4 | 0.594 |
| Medication use |  |  |  |
| Antihypertensive medication (%) | 50.2 | 30.7 | 0.594 |
| Lipid‐modifying medication (%) | 44 | 25.4 | 0.594 |
| Life style factors |  |  |  |
| Smoking, never/former/current (%) | 32.5/51.8/15.6 | 40.1/47.8/12.1 | 0.594 |
| Alcohol intake, none/low/high (%) | 21.1/56.8/22.2 | 17/59/24.1 | 0.594 |
| Brain MRI characteristics |  |  |  |
| Estimated total intracranial volume (mm3) | 1484492.6 ± 175461.1 | 1466803.8 ± 145372.4 | 0.033 |
| Brain volume (mm3) | 1165349.8 ± 120357.3 | 1183079.6 ± 115600.2 | 0.002 |
| MRI lag time (years) | 1.4 ± 1.4 | 1.2 ± 1.3 | 0.003 |

Data are presented as means ± standard deviation or percentage, and stratified for availability of MRI data. *Abbreviations*: NGM, normal glucose metabolism; T2D, type 2 diabetes; HbA1c, Hemoglobin A1c; HDL, high‐density lipoprotein; eGFR, estimated glomerular filtration rate; CVD, cardiovascular disease.

**Supplementary Table S3|** Associations of prediabetes and type 2 diabetes with hippocampal subfields

|  | Prediabetes | | Type 2 diabetes | |
| --- | --- | --- | --- | --- |
| **Structure** | **β±SE** | **p value** | **β±SE** | **p value** |
| THV | -0.036 ± 0.034 | 0.291 | **-0.151 ± 0.036** | **<0.001** |
|  |  |  |  |  |
| HATA | -0.041 ± 0.038 | 0.279 | -0.098 ± 0.040 | 0.015 |
| Fimbria | -0.085 ± 0.037 | 0.024 | **-0.195 ± 0.040** | **<0.001** |
| Fissure | -0.004 ± 0.040 | 0.923 | 0.026 ± 0.043 | 0.546 |
| Molecular layer | 0.024 ± 0.038 | 0.533 | -0.040 ± 0.040 | 0.316 |
| Dentate gyrus | -0.046 ± 0.034 | 0.179 | **-0.145 ± 0.037** | **<0.001** |
| CA4 | -0.039 ± 0.035 | 0.266 | **-0.113 ± 0.037** | **0.002** |
| CA2/3 | -0.058 ± 0.039 | 0.135 | **-0.155 ± 0.041** | **<0.001** |
| CA1 | 0.004 ± 0.036 | 0.916 | **-0.105 ± 0.038** | **0.006** |
| Subiculum | -0.021 ± 0.038 | 0.579 | **-0.134 ± 0.041** | **0.001** |
| Presubiculum | -0.048 ± 0.038 | 0.209 | **-0.118 ± 0.041** | **0.004** |
| Parasubiculum | 0.051 ± 0.040 | 0.204 | -0.005 ± 0.043 | 0.909 |
| Tail | -0.064 ± 0.040 | 0.109 | **-0.162 ± 0.042** | **<0.001** |

Results are presented as standardized beta coefficients (β) ± standard error (SE), and p-values. Bold shows multiple comparison correction significant (p<0.0071). Normal glucose metabolism is used as reference group. *Abbreviations*: THV, Total hippocampal volume; HATA, Hippocampus-amygdala-transition-area; CA, Cornu Ammonis.

**Supplementary Table S4|** Linear associations of glucose metabolism status as a continuum with hippocampal subfield volumes

| Structure | β±SE | p for trend |
| --- | --- | --- |
| THV | **-0.070 ± 0.017** | **<0.001** |
|  |  |  |
| HATA | -0.048 ± 0.019 | 0.014 |
| Fimbria | **-0.096 ± 0.019** | **<0.001** |
| Fissure | 0.011 ± 0.021 | 0.605 |
| Molecular layer | -0.014 ± 0.019 | 0.459 |
| Dentate gyrus | **-0.069 ± 0.018** | **<0.001** |
| CA4 | **-0.054 ± 0.018** | **0.002** |
| CA2/3 | **-0.075 ± 0.020** | **<0.001** |
| CA1 | -0.045 ± 0.018 | 0.014 |
| Subiculum | **-0.061 ± 0.020** | **0.002** |
| Presubiculum | **-0.058 ± 0.020** | **0.003** |
| Parasubiculum | 0.005 ± 0.021 | 0.826 |
| Tail | **-0.079 ± 0.020** | **<0.001** |

Results are presented as standardized beta coefficients (β) ± standard error (SE), and p-values. Bold shows multiple comparison correction significant (p<0.0071). *Abbreviations*: THV, Total hippocampal volume; HATA, Hippocampus-amygdala-transition-area; CA, Cornu Ammonis.

**Supplementary Table S5|** Associations of continuous measures of hyperglycemia with hippocampal subfield volumes

|  | Fasting plasma glucose | | 2 hour post load glucose | | HbA1c mmol/mol | |
| --- | --- | --- | --- | --- | --- | --- |
| Structure | β±SE | p value | β±SE | p value | β±SE | p value |
| THV | **-0.033 ± 0.010** | **0.001** | **-0.014 ± 0.004** | **<0.001** | **-0.005 ± 0.002** | **0.002** |
|  |  |  |  |  |  |  |
| HATA | -0.013 ± 0.011 | 0.259 | -0.006 ± 0.004 | 0.141 | -0.003 ± 0.002 | 0.160 |
| Fimbria | **-0.050 ± 0.011** | **<0.001** | **-0.022 ± 0.004** | **<0.001** | **-0.010 ± 0.002** | **<0.001** |
| Fissure | -0.003 ± 0.012 | 0.820 | 0.003 ± 0.004 | 0.557 | 0.002 ± 0.002 | 0.328 |
| Molecular layer | -0.011 ± 0.011 | 0.331 | -0.004 ± 0.004 | 0.361 | -0.002 ± 0.002 | 0.342 |
| Dentate gyrus | **-0.032 ± 0.010** | **0.002** | **-0.013 ± 0.004** | **<0.001** | -0.004 ± 0.002 | 0.007 |
| CA4 | -0.027 ± 0.010 | 0.010 | -0.010 ± 0.004 | 0.009 | -0.003 ± 0.002 | 0.053 |
| CA2/3 | **-0.039 ± 0.011** | **<0.001** | **-0.014 ± 0.004** | **<0.001** | -0.004 ± 0.002 | 0.020 |
| CA1 | -0.025 ± 0.011 | 0.019 | -0.009 ± 0.004 | 0.014 | -0.003 ± 0.002 | 0.090 |
| Subiculum | -0.020 ± 0.011 | 0.088 | **-0.011 ± 0.004** | **0.005** | -0.004 ± 0.002 | 0.043 |
| Presubiculum | -0.019 ± 0.011 | 0.097 | -0.010 ± 0.004 | 0.017 | -0.004 ± 0.002 | 0.027 |
| Parasubiculum | 0.001 ± 0.012 | 0.957 | 0.003 ± 0.004 | 0.430 | -0.001 ± 0.002 | 0.449 |
| Tail | **-0.036 ± 0.012** | **0.003** | **-0.016 ± 0.004** | **<0.001** | **-0.006 ± 0.002** | **<0.001** |

Results are presented as standardized beta coefficients (β) ± standard error (SE), and p-values. Bold shows multiple comparison correction significant (p<0.0071). *Abbreviations*: HbA1c, Hemoglobin A1c; THV, Total hippocampal volume; HATA, Hippocampus-amygdala-transition-area; CA, Cornu Ammonis.

**Supplementary Table S6|** Associations of continuous measures of hyperglycemia with hippocampal subfield volumes, after the exclusion of participants with type 2 diabetes

|  | Fasting glucose t0 | | Glucose t120 | | HbA1c mmol/mol | |
| --- | --- | --- | --- | --- | --- | --- |
| Structure | β±SE | p value | β±SE | p value | β±SE | p value |
| THV | -0.008 ± 0.026 | 0.755 | -0.008 ± 0.008 | 0.320 | 0.001 ± 0.003 | 0.786 |
|  |  |  |  |  |  |  |
| HATA | -0.033 ± 0.029 | 0.258 | -0.011 ± 0.009 | 0.224 | -0.002 ± 0.004 | 0.615 |
| Fimbria | -0.033 ± 0.029 | 0.261 | **-0.031 ± 0.009** | **<0.001** | -0.004 ± 0.004 | 0.244 |
| Fissure | 0.034 ± 0.031 | 0.270 | 0.005 ± 0.010 | 0.589 | 0.005 ± 0.004 | 0.185 |
| Molecular layer | 0.036 ± 0.029 | 0.214 | 0.010 ± 0.009 | 0.282 | 0.001 ± 0.004 | 0.765 |
| Dentate gyrus | -0.018 ± 0.027 | 0.505 | -0.008 ± 0.008 | 0.314 | 0.002 ± 0.003 | 0.622 |
| CA4 | -0.019 ± 0.027 | 0.491 | -0.005 ± 0.008 | 0.559 | 0.003 ± 0.003 | 0.363 |
| CA2/3 | -0.029 ± 0.030 | 0.334 | -0.016 ± 0.009 | 0.074 | 0.005 ± 0.004 | 0.220 |
| CA1 | 0.011 ± 0.028 | 0.705 | 0.003 ± 0.009 | 0.761 | 0.006 ± 0.004 | 0.078 |
| Subiculum | 0.020 ± 0.030 | 0.498 | 0.000 ± 0.009 | 0.986 | 0.001 ± 0.004 | 0.736 |
| Presubiculum | -0.023 ± 0.030 | 0.432 | -0.008 ± 0.009 | 0.372 | -0.008 ± 0.004 | 0.031 |
| Parasubiculum | -0.007 ± 0.031 | 0.824 | 0.010 ± 0.010 | 0.277 | -0.008 ± 0.004 | 0.050 |
| Tail | -0.033 ± 0.031 | 0.291 | -0.022 ± 0.009 | 0.022 | -0.002 ± 0.004 | 0.530 |

Results are presented as standardized beta coefficients (β) ± standard error (SE), and p-values. Bold shows multiple comparison correction significant (p<0.0071). *Abbreviations*: HbA1c, Hemoglobin A1c; THV, Total hippocampal volume; HATA, Hippocampus-amygdala-transition-area; CA, Cornu Ammonis.

**Supplementary Table S7|** Associations of glucose metabolism status with hippocampal subfield volumes, after replacing intracranial volume for total brain volume

|  | Prediabetes | | Type 2 diabetes | |
| --- | --- | --- | --- | --- |
| **Structure** | **β±SE** | **p value** | **β±SE** | **p value** |
| THV | -0.005 ± 0.031 | 0.864 | -0.052 ± 0.033 | 0.117 |
|  |  |  |  |  |
| HATA | -0.020 ± 0.036 | 0.590 | -0.024 ± 0.039 | 0.531 |
| Fimbria | -0.064 ± 0.036 | 0.081 | **-0.133 ± 0.039** | **<0.001** |
| Fissure | 0.004 ± 0.040 | 0.912 | 0.062 ± 0.043 | 0.149 |
| Molecular layer | 0.040 ± 0.037 | 0.272 | 0.028 ± 0.039 | 0.482 |
| Dentate gyrus | -0.020 ± 0.032 | 0.531 | -0.054 ± 0.034 | 0.114 |
| CA4 | -0.013 ± 0.032 | 0.697 | -0.021 ± 0.035 | 0.540 |
| CA2/3 | -0.032 ± 0.037 | 0.381 | -0.078 ± 0.039 | 0.047 |
| CA1 | 0.031 ± 0.034 | 0.364 | -0.017 ± 0.036 | 0.636 |
| Subiculum | 0.005 ± 0.037 | 0.893 | -0.049 ± 0.039 | 0.204 |
| Presubiculum | -0.023 ± 0.037 | 0.525 | -0.039 ± 0.039 | 0.322 |
| Parasubiculum | 0.068 ± 0.039 | 0.084 | 0.057 ± 0.042 | 0.176 |
| Tail | -0.038 ± 0.038 | 0.322 | -0.086 ± 0.041 | 0.034 |

Results are presented as standardized beta coefficients (β) ± standard error (SE), and p-values. Bold shows multiple comparison correction significant (p<0.0071). *Abbreviations*: THV, Total hippocampal volume; HATA, Hippocampus-amygdala-transition-area; CA, Cornu Ammonis.

**Supplementary Table S8|** Associations of glucose metabolism status with hippocampal subfield volumes, after changing waist for BMI

|  | Prediabetes | | Type 2 diabetes | |
| --- | --- | --- | --- | --- |
| **Structure** | **β±SE** | **p value** | **β±SE** | **p value** |
| THV | -0.035 ± 0.034 | 0.307 | **-0.147 ± 0.036** | **<0.001** |
|  |  |  |  |  |
| HATA | -0.037 ± 0.038 | 0.321 | -0.090 ± 0.040 | 0.024 |
| Fimbria | -0.084 ± 0.037 | 0.024 | **-0.192 ± 0.039** | **<0.001** |
| Fissure | 0.001 ± 0.040 | 0.975 | 0.038 ± 0.043 | 0.371 |
| Molecular layer | 0.026 ± 0.038 | 0.489 | -0.032 ± 0.040 | 0.424 |
| Dentate gyrus | -0.044 ± 0.034 | 0.197 | **-0.140 ± 0.036** | **<0.001** |
| CA4 | -0.037 ± 0.035 | 0.286 | **-0.108 ± 0.037** | **0.003** |
| CA2/3 | -0.060 ± 0.039 | 0.120 | **-0.158 ± 0.041** | **<0.001** |
| CA1 | 0.007 ± 0.036 | 0.855 | -0.098 ± 0.038 | 0.009 |
| Subiculum | -0.022 ± 0.038 | 0.571 | **-0.134 ± 0.040** | **<0.001** |
| Presubiculum | -0.048 ± 0.038 | 0.207 | **-0.117 ± 0.040** | **0.004** |
| Parasubiculum | 0.055 ± 0.040 | 0.170 | 0.003 ± 0.042 | 0.939 |
| Tail | -0.065 ± 0.040 | 0.104 | **-0.162 ± 0.042** | **<0.001** |

Results are presented as standardized beta coefficients (β) ± standard error (SE), and p-values. Bold shows multiple comparison correction significant (p<0.0071). *Abbreviations*: THV, Total hippocampal volume; HATA, Hippocampus-amygdala-transition-area; CA, Cornu Ammonis.

**Supplementary Table S9|** Associations of type 2 diabetes with hippocampal subfield volumes, after changing total-to-HDL cholesterol ratio for LDL cholesterol level

|  | Prediabetes | | Type 2 diabetes | |
| --- | --- | --- | --- | --- |
| **Structure** | **β±SE** | **p value** | **β±SE** | **p value** |
| THV | -0.028 ± 0.034 | 0.400 | **-0.136 ± 0.036** | **<0.001** |
|  |  |  |  |  |
| HATA | -0.039 ± 0.038 | 0.296 | **-0.092 ± 0.040** | **0.023** |
| Fimbria | **-0.076 ± 0.037** | **0.043** | **-0.175 ± 0.040** | **<0.001** |
| Fissure | -0.002 ± 0.040 | 0.970 | 0.035 ± 0.043 | 0.419 |
| Molecular layer | 0.028 ± 0.038 | 0.465 | -0.039 ± 0.040 | 0.336 |
| Dentate gyrus | -0.040 ± 0.034 | 0.240 | **-0.133 ± 0.037** | **<0.001** |
| CA4 | -0.034 ± 0.035 | 0.332 | **-0.104 ± 0.037** | **0.005** |
| CA2/3 | -0.051 ± 0.038 | 0.181 | **-0.141 ± 0.041** | **<0.001** |
| CA1 | 0.011 ± 0.036 | 0.750 | **-0.089 ± 0.038** | **0.020** |
| Subiculum | -0.014 ± 0.038 | 0.711 | **-0.120 ± 0.041** | **0.003** |
| Presubiculum | -0.043 ± 0.038 | 0.260 | **-0.107 ± 0.041** | **0.009** |
| Parasubiculum | 0.053 ± 0.040 | 0.185 | -0.010 ± 0.043 | 0.825 |
| Tail | -0.059 ± 0.040 | 0.137 | **-0.148 ± 0.043** | **<0.001** |

Results are presented as standardized beta coefficients (β) ± standard error (SE), and p-values. Bold shows multiple comparison correction significant (p<0.0071). *Abbreviations*: THV, Total hippocampal volume; HATA, Hippocampus-amygdala-transition-area; CA, Cornu Ammonis.

**Supplementary Table S10|** Associations of glucose metabolism status with hippocampal subfield volumes, with no exclusion of cases based on segmentation accuracy (additional cases n=451)

|  | Prediabetes | | Type 2 diabetes | |
| --- | --- | --- | --- | --- |
| **Structure** | **β±SE** | **p value** | **β±SE** | **p value** |
| THV | -0.030 ± 0.033 | 0.356 | **-0.172 ± 0.034** | **<0.001** |
|  |  |  |  |  |
| HATA | -0.040 ± 0.036 | 0.267 | **-0.128 ± 0.038** | **<0.001** |
| Fimbria | -0.056 ± 0.035 | 0.114 | **-0.224 ± 0.037** | **<0.001** |
| Fissure | -0.017 ± 0.038 | 0.651 | 0.021 ± 0.040 | 0.595 |
| Molecular layer | 0.006 ± 0.036 | 0.868 | -0.017 ± 0.038 | 0.656 |
| Dentate gyrus | -0.035 ± 0.033 | 0.291 | **-0.171 ± 0.035** | **<0.001** |
| CA4 | -0.031 ± 0.033 | 0.361 | **-0.139 ± 0.035** | **<0.001** |
| CA2/3 | -0.056 ± 0.037 | 0.124 | **-0.184 ± 0.038** | **<0.001** |
| CA1 | 0.010 ± 0.034 | 0.769 | **-0.125 ± 0.036** | **<0.001** |
| Subiculum | -0.012 ± 0.037 | 0.735 | **-0.158 ± 0.038** | **<0.001** |
| Presubiculum | -0.039 ± 0.036 | 0.288 | **-0.141 ± 0.038** | **<0.001** |
| Parasubiculum | 0.023 ± 0.039 | 0.545 | -0.000 ± 0.040 | 0.997 |
| Tail | -0.058 ± 0.038 | 0.127 | **-0.171 ± 0.039** | **<0.001** |

Results are presented as standardized beta coefficients (β) ± standard error (SE), and p-values. Bold shows multiple comparison correction significant (p<0.0071). *Abbreviations*: THV, Total hippocampal volume; HATA, Hippocampus-amygdala-transition-area; CA, Cornu Ammonis.
